# Thymosin Beta 4 Prevents Oxidative Stress by Targeting Antioxidant and Anti-Apoptotic Genes in Cardiac Fibroblasts

**DOI:** 10.1371/journal.pone.0026912

**Published:** 2011-10-25

**Authors:** Sandeep Kumar, Sudhiranjan Gupta

**Affiliations:** Division of Molecular Cardiology, Department of Molecular Medicine, College of Medicine, Texas A&M Health Science Center, Scott & White, Central Texas Veterans Health Care System, Temple, Texas, United States of America; Ohio State University Medical Center, United States of America

## Abstract

**Rationale:**

Thymosin beta-4 (Tβ4) is a ubiquitous protein with diverse functions relating to cell proliferation and differentiation that promotes wound healing and modulates inflammatory responses. The effecter molecules targeted by Tβ4 for cardiac protection remains unknown. The purpose of this study is to determine the molecules targeted by Tβ4 that mediate cardio-protection under oxidative stress.

**Methods:**

Rat neonatal fibroblasts cells were exposed to hydrogen peroxide (H_2_O_2_) in presence and absence of Tβ4 and expression of antioxidant, apoptotic and pro-fibrotic genes was evaluated by quantitative real-time PCR and western blotting. Reactive oxygen species (ROS) levels were estimated by DCF-DA using fluorescent microscopy and fluorimetry. Selected antioxidant and antiapoptotic genes were silenced by siRNA transfections in cardiac fibroblasts and the effect of Tβ4 on H_2_O_2_-induced profibrotic events was evaluated.

**Results:**

Pre-treatment with Tβ4 resulted in reduction of the intracellular ROS levels induced by H_2_O_2_ in the cardiac fibroblasts. This was associated with an increased expression of antioxidant enzymes Cu/Zn superoxide dismutase (SOD) and catalase and reduction of Bax/Bcl_2_ ratio. Tβ4 treatment reduced the expression of pro-fibrotic genes [connective tissue growth factor (CTGF), collagen type-1 (Col-I) and collagen type-3 (Col-III)] in the cardiac fibroblasts. Silencing of Cu/Zn-SOD and catalase gene triggered apoptotic cell death in the cardiac fibroblasts, which was prevented by treatment with Tβ4.

**Conclusion:**

This is the first report that exhibits the targeted molecules modulated by Tβ4 under oxidative stress utilizing the cardiac fibroblasts. Tβ4 treatment prevented the profibrotic gene expression in the *in vitro* settings. Our findings indicate that Tβ4 selectively targets and upregulates catalase, Cu/Zn-SOD and Bcl_2_, thereby, preventing H_2_O_2_-induced profibrotic changes in the myocardium. Further studies are warranted to elucidate the signaling pathways involved in the cardio-protection afforded by Tβ4.

## Introduction

Reactive oxygen species (ROS) plays an important role in regulating a variety of cellular functions, including gene expression, cell growth and cell death and has been implicated as one of the major contributors of cardiac damage in various cardiac pathologies [1], [2], [3]. Increased ROS levels can cause damage to nucleic acids, lipids, and proteins and can directly damage the vascular cells, cardiac myocytes and cardiac fibroblasts [3], [4]. Oxidative stress has been shown to be a precursor of cardiac apoptosis and has also been implicated in cardiac hypertrophy and fibrosis [5], [6], [7]. The declining protective enzymes and the reduced adaptive capacity to counter the oxidative stress cause activation of apoptotic death pathways [8]. Although, cells, tissues and organs utilize multiple layers of antioxidant defenses and damage removal, the heart is particularly more vulnerable to oxidative damage as it has a weak endogenous antioxidant defense system [9].

It has been suggested that an increased level of oxidative stress heart is primarily due to the functional uncoupling of the respiratory chain caused by inactivation of complex I in the mitochondria [10] or due to impaired antioxidant capacity, such as reduced activity of Cu/Zn superoxide dismutase (Cu/Zn-SOD) and catalase [6], or stimulation of enzymatic sources, including xanthine oxidase, cyclo-oxygenase, nitric oxide synthase, and non-phagocytic NAD(P)H oxidases [11]. Irrespective of the source of the stress stimuli involved, oxidative damage remains the main challenge and numerous efforts have been made to devise strategies to protect the heart against oxidative damage. Considerable attempts have been made in the recent decades to discover an “ideal” cardio-protective agent, which can abrogate the oxidative damage and maladaptive changes in the heart. In this pursuit, thymosin β4 (Tβ4) emerged as a powerful candidate.

Tβ4, a 43 amino acids, ubiquitous intracellular protein, bind to and sequester G- actin to modulate cell migration [12]. Recent studies implicated that Tβ4 had multiple diverse physiological and pathological functions, such as wound healing, angiogenesis, preserved cardiac function after myocardial infarction which essentially depends on cell migration [13], [14], [15]. Essentially, Tβ4 possesses cardiac tissue repair properties that cause epicardial cell migration, neovascularization and revascularization, and activation of cardiac progenitor cells in the heart [16], [17]. Apart from the cardiac repair properties, it has been demonstrated that Tβ4 protects human corneal epithelial cells and conjunctival cells from apoptosis after ethanol and hydrogen peroxide (H_2_O_2_) injury [18], [19], [20]. A few reports exhibit the role of Tβ4 in the myocardial infarction settings where it promotes endothelial and myocardial cell survival, cardiac cell migration, activation of integrin linked kinase (ILK) and activation of Akt/Protein Kinase B that resulted in improved cardiac function and reduction in scarring [14], [16], [21]. In this context, we have shown that Tβ4 also plays a crucial role in cardiac protection by enhancing the levels of PINCH-1-ILK-α-parvin components and promotes Akt activation while substantially suppressing NF-κB activation [22]. A recent report showed that Tβ4 upregulates anti-oxidative enzymes in human corneal cells against oxidative stress [23].

In this study, we used rat neonatal cardiac fibroblasts to study the effects of Tβ4 under oxidative stress. The rationale for using cardiac fibroblasts in this study originates from the fact that apart from being the most abundant cell type in the mammalian heart [24], [25], [26], the fibroblasts are intricately involved in myocardial development and cardiac protection under various stimuli [27], [28]. Recently, it has been shown that cardiac fibroblasts also undergo apoptosis under myocardial damage which can further aggravate the myocardial injury [29] and this can occur independent of the myocyte death [30]. The action of Tβ4 on cardiac fibroblasts under oxidative stress and its effects are largely unknown. It was for this reason we utilized the cardiac fibroblasts in the *in vitro* model to study the cardioprotective effects of Tβ4 under oxidative stress.

Here, we showed the effects of Tβ4 on ROS act ivy, antioxidant enzymes, anti-apoptotic and pro-fibrotic gene expression in preventing H_2_O_2_ induced oxidative stress in rat cardiac fibroblasts.

## Materials and Methods

### Reagents

3-(4,5-Dimethylthiazol-2-yl)-2,5-diphenyltetrazolium bromide (MTT), hydrogen peroxide (H_2_O_2_), dimethyl sulfoxide (DMSO), 5-Bromo-2-DeoxyUridine, (BrdU) all were purchased from Sigma Life Science (St. Louis, MO, USA). Thymosin β4 was purchased from ProSpec-Tany TechnoGene Ltd, (Rehovot, Israel). Dihydroethidium (DHE), 2′,7′-dichlorodihydrofluorescein diacetate (H_2_DCFH-DA), diaminofluorescein 2-diacetate (DAF-2DA), 3,3′-dihexyloxacarbocyanine iodide (DiOC_6_), and chloromethyl-X-rosamine (MitoTracker Red), ProLong Gold anti-fade mounting media (anti-fade) were purchased from Molecular Probes (Eugene, OR, USA). Primary antibody for Mn-SOD was purchased from Upstate/Millipore (Billerica, MA, USA); Cu/Zn-SOD from Assay Designs Inc., (Ann Arbor, MI, USA); catalase from Santa Cruz Biotechnology (Santa Cruz, CA, USA); collagen type I and collagen type III from Rockland Immunochemicals, (Gilbertsville, PA, USA); connective tissue growth factor (CTGF) from Abcam Inc. (Cambridge, MA, USA), Bax, Bcl_2_, caspase-3 and GAPDH were purchased from Cell Signaling Technology (Beverly, MA, USA). Protease inhibitor cocktail tablets were purchased from Roche GmbH, (Mann-heim, Germany). Dulbecco's Modified Eagle Medium (DMEM), non-essential amino acid cocktail, Antibiotic and anti-mycotic solution, insulin, transferrin and selenium (ITS), and fetal Bovine Serum (FBS) were all purchased from GIBCO, Invitrogen (Carlsbad, CA, USA).

### Cell culture and treatment

Primary cultures of cardiac fibroblasts were prepared from ventricles of 1–3 day-old Wistar rats as described [31], [32] and were plated at a field density of 2.5×10^4^ cells per cm^2^ on coverslips, 6- well plates, 60 mm culture dishes, or 100 mm dishes as required with DMEM containing 10% FBS. After 24 h, cells were serum deprived overnight before stimulation. The dose of 100 µM H_2_O_2_ did not show any toxic effect and damage to the cells and was therefore used throughout the study. Tβ4 was pretreated 2 h before the H_2_O_2_ challenge at a final concentration of 1 µg/mL which was based on the previous report [23]. The cardiac cell culture experiments were approved by Institutional Biosafety Committee (IBC) and IACUC, Texas A & M University, Temple, TX.

### Standardization of H_2_O_2_ dose on cardiac fibroblasts

To determine the optimal sub-lethal working concentration of H_2_O_2_ that is sufficient to induce oxidative stress but does not cause considerable cell death, the cell viability of cardiac fibroblasts under oxidative stress using a dose course of H_2_O_2_ (1 to 250 µM) was evaluated by MTT assay as described [33]. The absorbance was measured at 570 nm using a microplate reader (Molecular Devices, SpectraMax 250). The effect of Tβ4 (1 µg/mL) was assessed on the H_2_O_2_ challenge and the cytotoxicity curve was constructed and expressed as percentage cell viability compared to control. To rule out the possible direct interaction between MTT with H_2_O_2_ or Tβ4, the treatment media was aspirated before the addition of MTT solution.

### Quantification of intracellular ROS levels

Cardiac fibroblasts after respective treatments were incubated with 50 µM H_2_DCFH-DA at 37°C in the dark for 30 min as described [33]. To rule out the possible direct cleavage of non-fluorescent DCFH-DA to fluorescent DCF by the nonspecific intracellular esterases and extracellular esterases in the serum, the treatment media was completely removed before H_2_DCFH-DA staining. Cells were then harvested, washed with phosphate-buffered saline (PBS), and re-suspended in 50 mM HEPES buffer (5 mM HEPES, pH 7.4; 5 mM KCl, 140 mM NaCl, 2 mM CaCl_2_, 1 mM MgCl_2_ and 10 mM glucose) before their fluorescence intensities were acquired by fluorimetry (SpectraMaxPro, USA).

### Confocal Microscopy

For qualitative estimation of the levels of intracellular ROS, cells were seeded on coverslips in 6-well plates, treated and subsequently incubated with 50 µM H_2_DCFH-DA at 37°C in the dark for 30 min as previously described [33]. Cells were then fixed with 2% paraformaldehyde, washed 3 times with PBS, and mounted using anti-fade on glass slides and observed under confocal laser scanning microscope (Fluoview FV1000) fitted with a 488 nm argon ion laser. Images were acquired using the F10-ASW 1.5 Fluoview software (Olympus, Tokyo, Japan). Since, ROS induces generation of other ROS species and loss of mitochondrial membrane potential, we estimated the levels of other free radicals like superoxide and nitric oxide using fluorescent probes. For this, DHE, DAF-2DA and MitoTracker Red were used for estimation of superoxide (O_2_^.-^) radicals, nitric oxide (NO) and mitochondrial membrane potential (Δψm), respectively [33]. Image intensities from 15–20 fields of at least 3–4 confocal images of the same treatment group were quantified using ImageJ version 1.34 (NIH, Bethesda, USA) as described previously by Elia *et. al*.[34]

### Western blot analysis

For western blot analysis, cardiac fibroblasts were treated with or without Tβ4 for 2 h before treatment with 100 µM of H_2_O_2_. Cells were washed with PBS and cytosolic protein extracts were prepared using 1X Cell Lysis buffer (Santa Cruz Biotechnology, CA, USA) supplemented with protease inhibitor cocktail. Protein concentrations were determined using the Bradford assay (Bio-Rad) as per manufacturer's protocol. Aliquots of protein lysates (40 µg/lane) were separated on sodium dodecyl sulfate–10% polyacrylamide gels and western blotting was performed as described previously [31]. The primary antibodies used in this study include Mn-SOD, Cu/Zn-SOD, catalase, Bcl_2_, Bax, Caspase-3, Col-I, Col-III, CTGF and GAPDH. The detection was performed using chemiluminescence assay (Cell Signaling Technology, Beverly, MA). Membranes were exposed to x-ray film to observe the bands (Kodak, Rochester, NY). The quantification of each western blot was measured by densitometry as described previously [31]. The changes observed as a result of H_2_O_2_ stimulation were expressed as fold change while the changes observed as a result of Tβ4 treatment were depicted as percent increase or decrease unless otherwise noted.

### RNA isolation and quantitative real-time PCR (q-RT-PCR) analyses

Total RNAs from the cardiac fibroblasts were extracted using RNEasy kit (Qiagen, Stanford, Valencia, CA) as per the manufacturer's instructions. For real-time RT-PCR, 200 ng to 1 µg of total RNAs was reverse transcribed to cDNA using high capacity cDNA synthesis kit (Applied Bio systems, Foster City, CA, USA) following the manufacturer's protocols. Quantitative PCR was then carried out in a MX-3005 real-time PCR equipment (Stratagene, Cedar Creek, USA), using 2 µl (20 ng) of cDNA template, 1 nmol of primers, and 12.5 µl of iQ SYBR green supermix (Bio-Rad, Hercules, CA) in a total volume of 25 µl reaction. The primers used for the quantitative PCR are shown in Table 1. The cDNA were amplified with initial denaturation at 95°C for 10 min, followed PCR by 40 cycles of: 95°C 30 s, 60°C 30 s, 72°C 40 s and finally 1 cycle of melting curve following cooling at 40°C for 10 s. To confirm amplification specificity the PCR products from each primer pair were subjected to a melting curve analysis. Analysis of relative gene expression was done by evaluating q-RT-PCR data by 2^(-ΔΔCt)^ method as described by others [35], [36]. Each experiment was repeated at least three times and GAPDH or 18S was used as housekeeping gene for internal control. The changes observed as a result of H_2_O_2_ stimulation were expressed as fold change while the changes observed as a result of Tβ4 treatment were depicted as percent increase or decrease unless otherwise noted.

**Table 1:** List of quantitative real-time PCR used in the study.

Gene	Forward Primer	Reverse Primer
CTGF	ACTATGATGCGAGCCAACTGC	TGTCCGGATGCACTTTTTGC
Collagen 1	TGGCCTTGGAGGAAACTTTG	CTTGGAAACCTTGTGGACCAG
Fibronectin	TGCAGTGACCAACATTGATCGC	AAAAGCTCCCGGATTCCATCC
Mn-SOD	TGGACAAACCTGAGCCCTAA	GACCCAAAGTCACGCTTGATA
Cu/Zn-SOD	TGGGAGAGCTTGTCAGGTG	CACCAGTAGCAGGTTGCAGA
Catalase	ATCAGGGATGCCATGTTGTT	GGGTCCTTCAGGTGAGTTTG
Bcl_2_	GTACCTGAACCGGCATCTG	GGGGCCATATAGTTCCACAA
BAX	CGAGCTGATCAGAACCATCA	GGGGTCCCGAAGTAGGAA
GAPDH	CCAGGTGGTTCCTCTGACTTC	GTGGTCGTTGAGGGCAATG
18S	TGTTCACCATGAGGCTGAGATC	TGGTTGCCTGGGAAAATCC

### RNA interference and siRNA transfection

Silencing of Cu/Zn-SOD, catalase and Bcl_2_ proteins was achieved by using small interfering RNA (si-RNA) transfections. Pre-designed double-stranded si-RNA against Cu/Zn-SOD (SASI_Rn01_00112871), catalase (SASI_Rn01_00053417) and Bcl_2_ (SASI_Rn01_00062026) were purchased from Sigma Life Science (Saint Louis, MO, USA). As a negative control, non-targeting control si-RNA (scrambled si-RNA), verified to have no significant effect on most essential mammalian genes, was obtained from Sigma. Rat neonatal fibroblasts were seeded into 6-well plates with 3 mL of complete DMEM. After 24 h, the medium was replaced with 2 mL of Opti-MEM (Life Technologies). Cells were then transfected with 200 pmol of the siRNAs for catalase, Cu/Zn-SOD, Bcl_2_ or negative control siRNA using N-TER™ nanoparticle siRNA transfection system (Sigma) as per the manufacturer's protocol. After 24 h of transfection, cells were treated and harvested to determine the transfection efficiency and effect of Tβ4 in presence and absence of H_2_O_2_ treatment. Quantification of caspase-3 gene expression by q-RT- PCR was done to study the extent of pro-apoptotic gene expression.

### TUNEL staining

Quantification of TUNEL staining was done to study the extent of apoptotic cell death on transfected fibroblasts by *in situ* cell death detection kit (Roche Applied Science, Indianapolis, IN). Briefly, 2×10^5^ fibroblasts were seed on the coverslips in the 6-well plates, followed by siRNA transfections and treatment. The TUNEL staining was done using deoxyneucleotidyl transferase (TdT) and FITC-labeled 2′-deoxyuridine 5′-triphosphate (dUTP) as per manufacturer's instructions. The Analysis was done using 3–5 images from hi-power field (40X objective) and the TUNEL index (%) was calculated as the number of TUNEL-positive cells divided by the total number of DAPI-stained nuclei.

### Statistical analyses

All experiments were performed at least three times for each determination. Data are expressed as means±Standard Error (SE) and were analyzed using student t-test or one-way analysis of variance (ANOVA) and secondary analysis for significance with Tukey–Kramer post tests using Prism 5.0 Graph Pad software (Graph Pad, San Diego, CA, USA). A p value less than 0.05 was considered statistically significant.

## Results

### Pretreatment with Tβ4 improves cardiac fibroblasts survival

**Figure 1:**
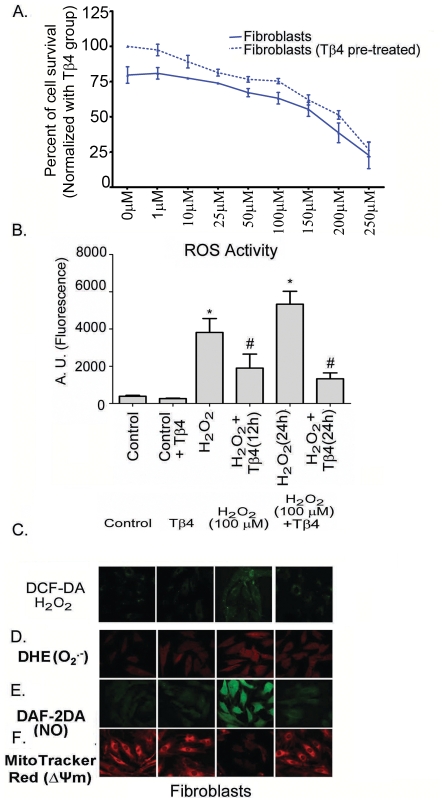
Effect of Tβ4 on cell viability in H_2_O_2_ treated fibroblasts. (**A**) The MTT assay was performed with increasing H_2_O_2_ concentration (1 to 250 µM) in presence (dotted lines) and absence (solid lines) of Tβ4 (1 µg/mL). Data represent means±SEM of 3 individual experiments. (**B**) Representative confocal laser scanning microscopy images of cardiac fibroblasts stained with DCF-DA showing the effect of Tβ4 on intracellular ROS upon treatment with H_2_O_2_. (**C**). Effect of Tβ4 on generation of ROS in fibroblasts treated with H_2_O_2_ by fluorimetry. The graph represents the percentage of fluorescence positive fibroblasts upon staining with DCF-DA. Data represent the mean±SE of at least three separate experiments. * means p<0.05 compared to the controls and # represents p<0.05 compared to the respective H_2_O_2_ treated group (**D**) Representative confocal laser scanning microscopy images of cells stained with DHE Red showing the effect of Tβ4 on generation of superoxide radicals upon treatment with H_2_O_2_ in fibroblast. (**E**). Representative confocal laser scanning microscopy images of cells stained with DAF-2DA showing the effect of Tβ4 on generation of nitric oxide upon treatment with H_2_O_2_ in fibroblast. (**F**). Representative confocal laser scanning microscopy images of cells stained with Mitotracker Red showing the effect of Tβ4 on loss of mitochondrial membrane potential upon treatment with H_2_O_2_ in fibroblast.

The viability of cardiac fibroblasts cells was assessed after treating the cells with various concentrations of H_2_O_2_ for 24 h by MTT assay. We observed that the 50% lethal dose (LD_50_) of H_2_O_2_ on these cells was between 150 and 250 µM (Figure 1A). Pretreatment with Tβ4 (1 µg/mL) resulted in an improved cell survival count (19.2%; p<0.05) compared to the untreated group. This beneficial effect of pretreatment was observed until 100 uM H_2_O_2_ concentration after which the pretreatment effect of Tβ4 was compromised. This indicated a potential role of Tβ4 in protecting the cardiac fibroblasts against cell death due to generation of ROS. The optimal concentration of H_2_O_2_ was determined from MTT experiment and thus standardized dose of 100 µM H_2_O_2_ which was used throughout the study.

### Tβ4 protects cardiac fibroblasts against H_2_O_2_ induced oxidative stress

Oxidative stress leads to detrimental changes in the cell signaling process. To determine whether Tβ4 has any effect on ROS activity under oxidative stress, intracellular ROS levels in the cardiac fibroblast were estimated after H_2_O_2_ treatment by confocal microscopy and fluorimetry. Cardiac fibroblasts treated with H_2_O_2_ showed enhancement of fluorescence intensity of DCF-DA by 9.9-fold at 12 h (p<0.01) and 13.8-fold at 24 h (p<0.01), respectively, compared to the untreated controls (Figure 1B). Pretreatment with Tβ4 showed a 50.2% decrease at 12 h (p<0.05) and 75.1% at 24 h (p<0.05), respectively, compared to H_2_O_2_ treated cardiac fibroblasts. Tβ4 treatment reduced the DCF-DA fluorescence by 32.2% (p<0.01) in the unstimulated cardiac fibroblast indicating that Tβ4 treatment was effective in preventing oxidative stress in cardiac fibroblasts. The confocal microscopy images matched with the quantitative fluorimetry results (Figure 1C). Our data indicated that Tβ4 abrogated the ROS activity significantly in cardiac fibroblasts.

### Tβ4 reduces the formation of superoxide radicals and nitric oxide

Oxidative stress generated superoxide and nitric oxide radicals. These can lead to production of more noxious free radicals, like peroxynitrite (ONOO^-^). To evaluate the effect of Tβ4 in superoxide and nitric oxide radical generation in cardiac fibroblasts, we estimated the levels of those free radical generations from H_2_O_2_ treatment by using confocal microscopy. The confocal microscopy data showed that H_2_O_2_ treated cells enhanced the fluorescence intensity of DHE and DAF-2DA, an indicator of increased O_2_^.-^ and NO radicals, compared to the controls (Figure 1D and E). The enhanced fluorescence intensity of DHE and DAF-2A was prevented by Tβ4 pretreatment, compared to the H_2_O_2_ treated cells (Figure 1 D and E). The quantifications of image intensities were tabulated in Table 2. Together, our data suggest that Tβ4 attenuated H_2_O_2_ induced superoxide and nitric oxide radical generation in cardiac fibroblasts.

**Table 2:** Image intensities (Arbitrary Units) showing the fluorescence intensities in cardiac fibroblasts upon staining with DCF-DA, DHE, DAF-2DA and MitoTracker Red.

S. No.	Staining	Control	Tβ4	H_2_O_2_	H_2_O_2_ + Tβ4
1	DCF-DA (H_2_O_2_)	259± 48	96±19*	614±26*	183±41#
2	DHE (O_2_ **^-^** radicals)	428±52	461±81	892±62*	659±35#
3	DAF-2DA (NO)	305±43	274±43	948±47*	308±41#
4	MitoTracker Red (Δψm)	644±58	657±73	253±45*	618±38#

Data acquired from at least 15 fields taken from 3–4 different confocal images of the same treatment group and were quantified by using ImageJ Software.

*denotes p<0.05 compared to controls while.

# denotes p<0.05, compared to the H_2_O_2_-treated group.

### Tβ4 treatment prevents the loss of mitochondrial membrane potential (Δψm)

Oxidative stress impacts the mitochondrial function leading to disruption of electron transport and loss of mitochondrial transmembrane potential. We evaluated the effect of Tβ4 on the mitochondrial membrane potential in the cardiac fibroblast under H_2_O_2_ induced oxidative stress using MitoTracker® Red by confocal microscopy. Our data showed that there was loss of Δψm as indicated by a decrease in the fluorescence intensity of MitoTracker® Red in H_2_O_2_-treated cells. This decrease in the fluorescence intensity was prevented by the pretreatment with Tβ4 (Figure 1 F) suggesting that Tβ4 protected mitochondrial function by preventing the loss of Δψm in the cardiac fibroblast. The quantifications of image intensities were tabulated in Table 2. Our data suggest that Tβ4 treatment significantly reduced mitochondrial potential in H_2_O_2_ treated cardiac fibroblasts.

### Tβ4 selectively upregulates the expression of antioxidant genes in cardiac fibroblasts under oxidative stress

**Figure 2:**
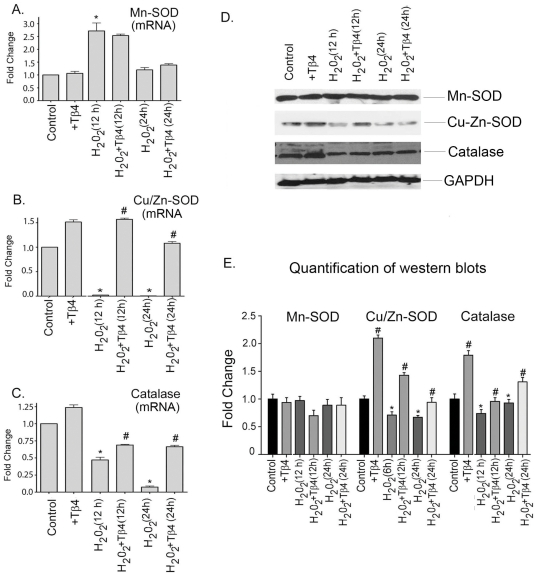
Effect of Tβ4 on antioxidant genes under oxidative stress in cardiac fibroblasts. Cells were treated with H_2_O_2_ in presence and absence of Tβ4 and (**A**) Mn-SOD, (**B**) Cu/Zn-SOD and (**C**) catalase, mRNA expression were analyzed at 12 h and 24 h, respectively by qRT-PCR. Data represent the means±SE of at least three separate experiments. (**D**) Western blot analysis showed the protein expression of Mn-SOD, Cu/Zn-SOD and catalase at 12 h and 24 h, respectively. GAPDH was used as internal loading control for the experiment. (**E**) Graph shows the relative fold change in the protein expression of Mn-SOD, Cu/Zn-SOD and catalase, respectively by densitometry. Data represent means±SEM from 3 individual experiments. * denotes p<0.05, compared to controls while^ #^ denotes p<0.05, compared to the H_2_O_2_-treated group.

We examined the effect of Tβ4 on mRNA expression of antioxidant genes like Mn-SOD, Cu/Zn-SOD and catalase in cardiac fibroblasts under oxidative stress by q-RT- PCR. Our data showed that there was an increase in the expression of Mn-SOD in the H_2_O_2_-treated cells by 2.7-fold and 1.3-fold increase at 12 and 24 h, respectively, compared to the untreated cells. Tβ4 treatment did not alter the mRNA expression of Mn-SOD at these time points, compared to the H_2_O_2_-treated groups (Figure 2A). H_2_O_2_ treatment reduced the mRNA expression of Cu/Zn-SOD by 95% at 12 h (p<0.05) and 98% at 24 h (p<0.05), respectively, compared to the untreated cells. Treatment with Tβ4 resulted in an increase of expression of Cu/Zn-SOD. Tβ4 pretreatment restored the Cu/Zn-SOD mRNA expression back to normal levels both at 12 h and 24 h, respectively, compared to the H_2_O_2_ treated cells (Figure 2B). In unstimulated cell, the mRNA expression of catalase was upregulated by 1.3-fold after Tβ4 treatment. H2O2 treatment reduces the catalase mRNA expression by 50% (p<0.05) and 89% (p<0.05) at 12 and 24 h treatment, respectively, compared to the control. Pretreatment with Tβ4 upregulated the catalase mRNA expression by 1.5-fold (p<0.05), and 6.4-fold (p<0.05) at 12 h and 24 h, respectively, compared to the H_2_O_2_ treated cells (Figure 2C).

To examine the status of the above antioxidant genes at the translational levels, western blots were performed. Our data showed that the expression level of Mn-SOD was relatively unchanged at protein level at 12 h and 24 h, respectively. Treatment with Tβ4 did not alter the expression of Mn-SOD (Figure 2D). Compared to the untreated cells, H2O2 treatment showed a decline of Cu/Zn-SOD protein expression by 23% (p<0.05) and 24.6% (p<0.05) at 12 h and 24 h, respectively, compared to the controls. Cells pretreated with Tβ4 showed a 2.0-fold (p<0.05) and 1.4-fold (p<0.05) increased in the expression of Cu/Zn-SOD at 12 h and 24 h treatment, respectively, compared to the H_2_O_2_ treated cells (Figure 2D). The expression of catalase declined by 25% (p<0.05) at 12 h and 15% (p<0.05) at 24 h, respectively, with H_2_O_2_ treatment compared to untreated cells. Tβ4 pretreatment increased catalase expression by 1.3-fold (p<0.05) and 1.4-fold (p<0.05) increase at 12 h and 24 h, respectively, compared to the H_2_O_2_ treated cells (Figure 2D). The normalized quantification for Mn-SOD, Cu/Zn-SOD and catalase expression by densitometry is shown in the Figure 2E.

### Tβ4 promotes cell survival by increasing the expression of anti-apoptotic genes and reducing the expression pro-apoptotic genes in cardiac fibroblasts under oxidative stress

**Figure 3:**
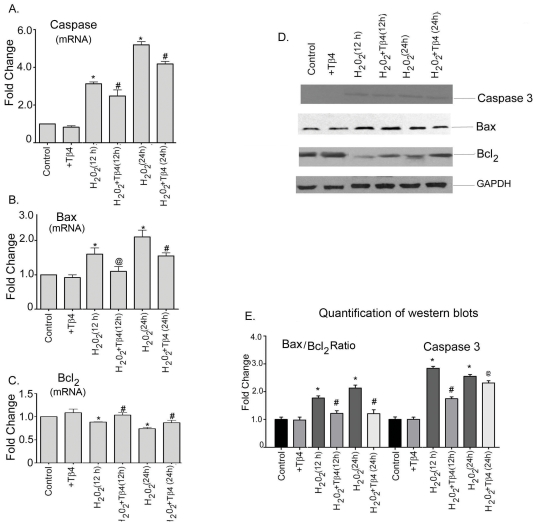
Effect of Tβ4 on pro- and anti-apoptotic genes under oxidative stress in cardiac fibroblasts. Cells were treated with H_2_O_2_ in the presence and absence of Tβ4 and (**A**) Caspase-3, (**B**) Bax and (**C**) Bcl_2_ mRNA expression were analyzed at 12 h and 24 h, respectively by qRT-PCR. Data represent the means±SEM of at least three separate experiments. (**D**) Protein expression of Caspase-3, Bax and Bcl_2_ at 12 h and 24 h, respectively. GAPDH was used as loading control for the experiment. (**E**) GAPDH was used as internal loading control for the experiment. (**E**) Graph shows the relative fold change in the protein expression of Bax, Bcl_2_ and caspase-3, respectively by densitometry. Data represent means±SEM from 3 individual experiments. * denotes p<0.05 compared to controls while^ #^ denotes p<0.05, compared to the H_2_O_2_-treated group and ^@^ means p = ns compared, to the H_2_O_2_-treated group.

To examine the effect of Tβ4 on cardiac death and survival, we determine the expression of caspase-3, Bax (pro-apoptotic) and Bcl_2_ (anti-apoptotic) in presence and absence of H_2_O_2_ by qRT-PCR. H_2_O_2_ treatment upregulated the caspase-3 expression by 3.0-fold (p<0.05) and 5.0-fold (p<0.05) at 12 h and 24 h treatment, respectively, compared to the untreated cells. Tβ4 pretreatment resulted in 29% (p<0.05) and 19% (p<0.05) decrease in the mRNA expression of caspase-3 at 12 h and 24 h treatment, compared to H_2_O_2_ treated cells (Figure 3A). H_2_O_2_ treatment increased mRNA expression of Bax by 1.6-fold (p<0.05) and 2.1-fold (p<0.05) at 12 h and 24 h, respectively, compared to untreated cells. The mRNA expression of Bax was reduced by 31% (p<0.05) and 26% (p<0.05) at 12 h and 24 h, respectively with pretreatment Tβ4 treatment (Figure 3B). The mRNA expression of Bcl_2_ concomitantly decreased by 11% (p<0.05) and 23% (p<0.05) at 12 h and 24 h, respectively in H_2_O_2_ treatment. The mRNA levels of Bcl_2_ was increased by pretreatment with Tβ4 by 20% (p<0.05) and 14% (p<0.05) at 12 h and 24 h, respectively, compared to H_2_O_2_-treated cells (Figure 3 C).

At translational level, H_2_O_2_ treatment resulted in a 2.8-fold (p<0.05) and 2.6-fold (p<0.05) increase in the expression of caspase-3 protein at 12 h and 24 h period, respectively, compared to the untreated cells. Tβ4 pretreatment showed 39% (p<0.05) and 9% (p = ns) decrease in the caspase-3 expression at the above time point, compared to H_2_O_2_-treated cells. (Figure 3 D). GAPDH was used as internal loading control. The normalized quantification of caspase-3 by western blotting is shown in the Figure 3E. H_2_O_2_ treatment showed an increase in the Bax/Bcl_2_ ratio by 1.8-fold (p<0.05) and 2.3-fold (p<0.05) at 12 h and 24 h, respectively, compared to the controls. Tβ4 treatment significantly prevented this increase in the Bax/Bcl_2_ ratio by 31% (p<0.05) and 43% (p<0.05) at 12 h and 24 h, respectively, compared to H_2_O_2_-treated cells (Figure 3 E).

### Tβ4 reduced the expression of pro-fibrotic genes in cardiac fibroblast under oxidative stress

**Figure 4:**
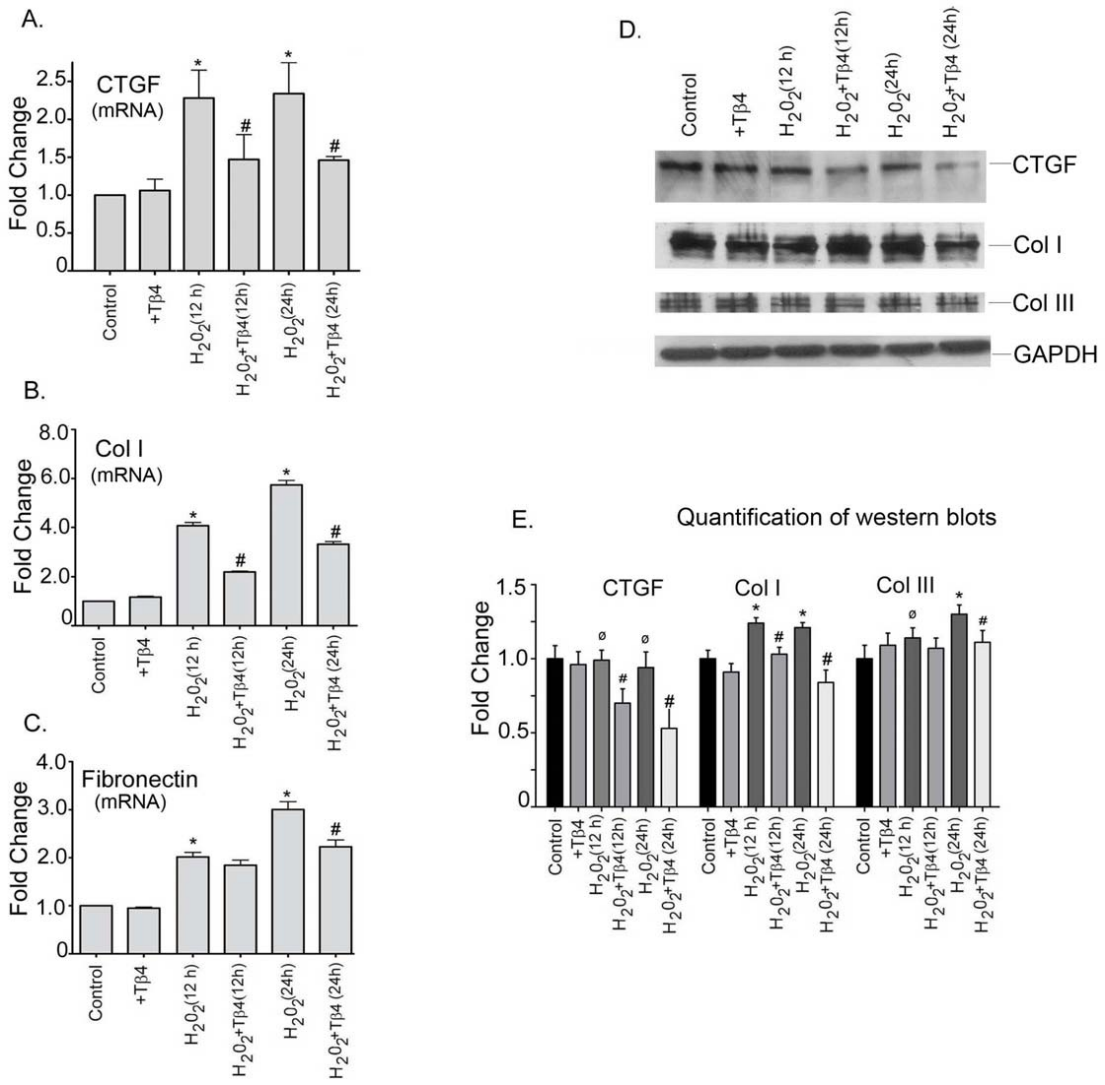
Effect of Tβ4 on profibrotic genes under oxidative stress in cardiac fibroblasts. Cells were treated with H_2_O_2_ in the presence and absence of Tβ4 and (**A**) CTGF, (**B**) Col-I and (**C**) Fibronectin mRNA expression was analyzed at 12 h and 24 h, respectively by qRT-PCR. Data represent the means±SE of three separate experiments. (**D**) Western blot analysis showed the protein expression of CTGF, Col-I and Col-III at 12 h and 24 h, respectively. GAPDH was used as a loading control for the experiment. (**E**) Graph shows the relative fold change in the protein expression of Bax, Bcl_2_ and caspase-3, respectively by densitometry. Data represent means±SEM from 3 individual experiments. * denotes p<0.05, compared to controls while^ #^ denotes p<0.05 compared to the H_2_O_2_-treated group and ø denotes p>0.05, compared to controls.

We next determine the pro-fibrotic gene expression under oxidative stress condition. Our data showed that H_2_O_2_ treatment increased the mRNA expression of CTGF by 2.3-fold (p<0.05) and 2.3-fold (p<0.05) at 12 h and 24 h, respectively, compared to the controls. Tβ4 treatment showed reduction in the mRNA expression of CTGF by 17% (p<0.05) and 38% (p<0.05) at 12 h and 24 h, respectively (Figure 4 A). The mRNA expression of collagen-1 was increased by 4.0-fold (p<0.05) and 5.7-fold (p<0.05) at 12 h and 24 h, respectively, in H_2_O_2_-treated cells compared to the controls. Tβ4 pretreatment decreased collagen-I by 46% (p<0.05) and 42% (p<0.05) at 12 h and 24 h, respectively, compared to the H_2_O_2_-treated cells (Figure 4 B). The mRNA expression of fibronectin was increased by 2.1-fold (p<0.05) and 3.3-fold (p<0.05) at 12 h and 24 h, respectively, in the H_2_O_2_-treated cells. The mRNA expression of fibronectin in the Tβ4 treatment was decreased by 9% (p = ns) and 34% (p<0.05) at 12 h and 24 h, respectively, compared to the H_2_O_2_-treated cells (Figure 4C).

At translational level, we did not observe any significant change in the CTGF protein level under oxidative stress. However, pretreatment with Tβ4 reduced the expression of CTGF by 27% (p<0.05) and 43% (p<0.05) at 12 h and 24 h, respectively, compared to the H_2_O_2_ treated cells. The collagen-I protein expression also increased in H_2_O_2_ treatment by 24% (p<0.05) and 21% (p<0.05) at 12 h and 24 h treatment, respectively, compared to the controls. Tβ4 treatment reduced collagen-I protein expression by 17% (p<0.05) and 30.5% (p<0.05) at 12 h and 24 h, respectively, compared to the H_2_O_2_ treated cells. The collagen-III protein expression also increased in H_2_O_2_ treatment by 14% (p<0.05) and 30% (p<0.05) at 12 h and 24 h, respectively, compared to controls. Tβ4 treatment reduced the collagen-III expression by 7% (p = ns) and 16% (p<0.05) at 12 h and 24 h, respectively, compared to the H_2_O_2_ treated cells (Figure 4 D). The normalized quantification of each western blot profile is shown (Figure 4 E).

### Effect of knocking down of Cu/Zn-SOD, catalase and Bcl_2_ in cardiac fibroblasts

We first determine the efficacy of siRNA mediated transfection of Cu/Zn-SOD, catalase and Bcl_2_ in cardiac fibroblasts. Western blots were performed to confirm the specific knock-down of Cu/Zn-SOD, catalase and Bcl_2_ in cardiac fibroblasts.

**Figure 5:**
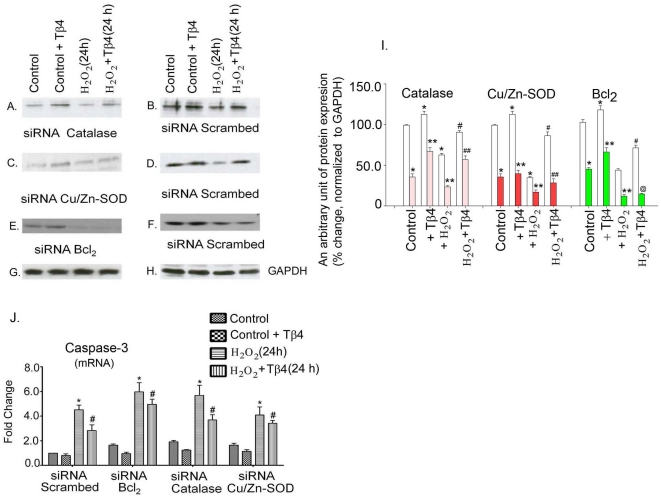
Effect of Tβ4 on antioxidant and antiapoptotic genes under siRNA knock down of catalase, Cu/Zn-SOD and Bcl_2_ genes in cardiac fibroblasts under normal and oxidative stress condition. Representative western blot illustrating the restoration of the expression of catalase, Cu/Zn-SOD and Bcl_2_ in the cardiac fibroblasts upon knockdown of antioxidant and antiapoptotic genes, viz., (**A**) siRNA-catalase *vs*. (**B**) scrambled siRNA; (**C**) siRNA-Cu/Zn-SOD *vs*. (**D**) scrambled siRNA and (**E**) siRNA-Bcl_2_ *vs*. (**F**) scrambled siRNA, respectively. (**G and H**) GAPDH was used as an internal loading control for the above experiments. (**I**) Effect of Tβ4 on antioxidant and antiapoptotic genes under control and siRNA knock down of catalase, Cu/Zn-SOD and Bcl_2_ genes in cardiac fibroblasts under normal and oxidative stress condition. Data represent the means±SE of at least three separate experiments. Clear bars represent the normalized protein expression of respective proteins in the si-scrambled RNA condition while the color bars represent the normalized protein expression under the respective siRNA knockdown conditions. * denotes p<0.05, compared to si-scramble control, ^#^ denotes p<0.05, compared to si-scramble and H_2_O_2_ treated group, ** denotes p<0.05, compared to si-RNA control group and ^##^ denotes p<0.05, compared to the respective si-RNA and H_2_O_2_ treated group while ^@^ denotes p = ns, compared to si-RNA and H_2_O_2_ treated group. (**J**) Bar graph shows relative fold-change in the mRNA expression of caspase-3 in Tβ4 pretreated cardiac fibroblasts under respective siRNA knockdown and H_2_O_2_ induced oxidative stress. Data represent the means±SE of at least three separate experiments. * denotes p<0.05, compared to controls while^ #^ denotes p<0.05 compared to the H_2_O_2_-treated group.

To determine whether Tβ4 has target for Cu/Zn-SOD, catalase and Bcl_2_, we tested the effect of Tβ4 in the cardiac fibroblasts either transfected with scrambled siRNA or siRNA of catalase, Cu/Zn-SOD, and Bcl_2_ in the presence and absence of H_2_O_2_ induced oxidative stress. Treatment with scrambled siRNA did not alter the expression of catalase, Cu/Zn-SOD, and Bcl_2_; therefore, scrambled siRNA treated group was taken as control for this experiment. Cardiac fibroblasts transfected only with scrambled siRNA showed 15.5±5.4%, 12.6±6.4% and 18.1±0.9% (p<0.05) increased protein expression of catalase, Cu/Zn-SOD and Bcl_2_, due to Tβ4 treatment (Fig 5 B, D and E, 2^nd^ lane). H_2_O_2_ treatment under non-silencing (scramble treated) conditions resulted in 38.4±4.2%, 63.3±3.1% and 56.4±4.6% (p<0.05) down regulation of catalase, Cu/Zn-SOD, and Bcl_2_, respectively, compared to the control (Fig 5 B, D and E, 3^rd^ lane vs. 1^st^ Lane). In the scramble treated condition, Tβ4 pre-treatment resulted in significant upregulation of catalase, Cu/Zn-SOD, and Bcl_2_ (44.6±3.2%, 145.6±11.2%, 62.1±5.7%; respectively, p<0.05), compared to H_2_O_2_ treated cardiac fibroblasts (Fig 5 B, D and E, 4^th^ lane).

In comparison to the scrambled siRNA treated set (Fig 5B, D and F, 1^st^ lane), our data showed 64.2±3.1% (p<0.05) knock-down of catalase, 77.1±2.9% (p<0.05) knock-down of Cu/Zn-SOD and 61.2±3.9% (p<0.05) knock-down of Bcl_2_, when their specific siRNAs were transfected into the fibroblasts (Figure 5A, C and E, 1^st^ lane).

The down regulation in the levels of catalase, Cu/Zn-SOD and Bcl_2_ proteins was abrogated by pre-treatment of Tβ4 (Fig 5 A, C and E, 2^nd^ lane). We observed that the expression of catalase, Cu/Zn-SOD, and Bcl_2_ was upregulated by 84.5±5.4%, 45.6±11.2%, 39.8±6.4%, respectively, p<0.05, compared to their control group (Fig 5 A, C and E, 1^st^ lane). Furthermore, to evaluate the effect of Tβ4 under more stringent conditions, the siRNA-treated fibroblasts were subjected to H_2_O_2_ challenge, to further enhance the levels of oxidative stress. Our data showed that H_2_O_2_ treatment further depleted the levels of catalase, Cu/Zn-SOD and Bcl_2_ (Figure 5A, C, E (3^rd^ Lane). This dual stress ultimately reduced the expression of catalase, Cu/Zn-SOD and Bcl_2_ to 23.3±3.8%, 17.2±4.1% and 12.7±2.1% (p<0.05) of the endogenous levels (Figure 5A, C, E; 3^rd^ Lane). Tβ4 pretreatment resulted in a significant upregulation of catalase and Cu/Zn-SOD (142.4±7.3% and 36.5±4.1%, respectively, p <0.05) but not for Bcl_2_ (12.5±8.1%, p>0.05), compared to respective controls (Figure 5A, C, E (4^th^ Lane). This implicates that under conditions Tβ4 restored the levels of catalase and Cu/Zn-SOD but failed to restore the expression of Bcl_2_. GAPDH was used as an internal loading control (Figure 5 G and H). The quantification of knock down of catalase, Cu/Zn-SOD and Bcl_2_ protein and the restoration of their expression upon Tβ4 treatment is shown in Fig 5 I.

To test the effect of Tβ4 on induction of apoptosis, we further determined the expression of caspase-3, under the similar condition stated above. In scramble transfected cells, the expression of caspase-3 increased to 3.8-fold (p<0.05) in H_2_O_2_ treatment, which reduced by 26% (p<0.05) upon pretreatment with Tβ4 (Figure 5 J). In Bcl_2_ knocked down cells, the expression of caspase-3 increased by 1.7-fold (p<0.05), compared to the scrambled siRNA-transfected cells. H_2_O_2_ treatment potentiate a 6.0-fold (p<0.05) increase of caspase-3, suggesting an additive effect. Tβ4 treatment resulted in an 18% reduction (p<0.05) in the expression of caspase-3, compared to H_2_O_2_ stimulated cells (Figure 5J, second group). Similarly, knockdown of catalase gene resulted an increase of 2.1-fold (p<0.05) caspase-3 expression. H_2_O_2_ treatment showed an additive effect by increasing a 5.7-fold (p<0.05) of caspase-3. Tβ4 pretreatment resulted in a 25% (p<0.05) reduction in the caspase-3 activity in the H_2_O_2_ stimulated cells (Figure 5J, third group), compared to the H_2_O_2_-treated group. The expression of caspase-3 also increased by 1.5-fold (p<0.05) when Cu/Zn-SOD was knocked down in fibroblasts. H_2_O_2_ treatment under similar condition resulted in 4.1fold (p<0.05) increase of caspase-3 and Tβ4 pretreatment attenuated the caspase-3 activity by 18% (p<0.05), compared to H_2_O_2_ treated cells (Figure 5J, fourth group).

**Figure 6:**
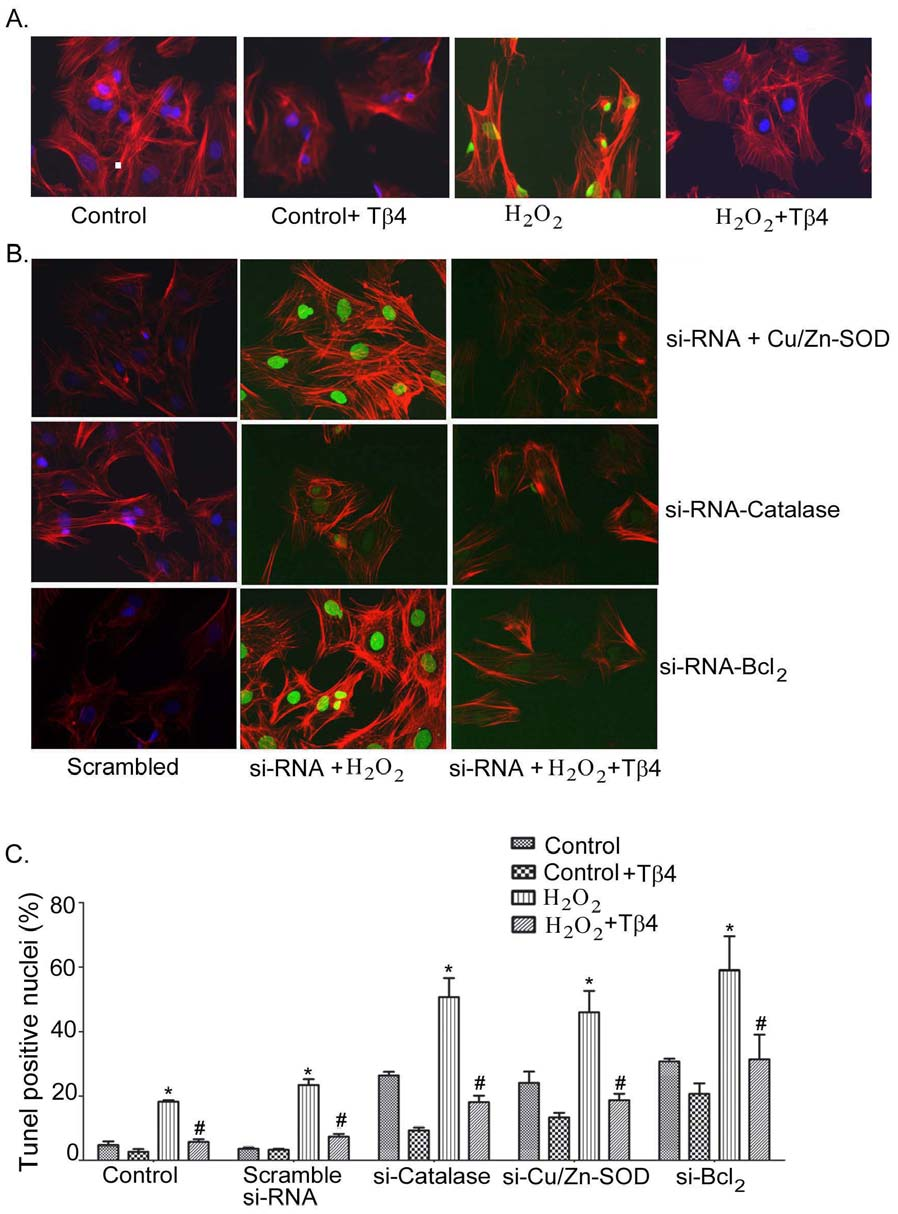
Effect of Tβ4 on cardiac fibroblast apoptosis under oxidative stress. (**A**) Representative fluorescent microscopy images of TUNEL staining in rat neonatal cardiac fibroblasts. Bright TUNEL-positive staining (FITC) was observed in H_2_O_2_ treatment which was not observed in control cells and cells pretreated with Tβ4. DAPI was used to stain the intact nuclei and counterstaining of filamentous actin was done with Texas Red®-X phalloidin. (**B**) Representative fluorescent microscopy images showing the effect of Tβ4 treatment in presence and absence H_2_O_2_-induced oxidative stress on cardiac fibroblasts transfected with siRNAs of Cu/Zn-SOD, catalase and Bcl_2_ *vs*. scrambled siRNA, respectively. (**C**) Bar graph shows the percent TUNEL-positive nuclei under similar experimental condition. Data represent the means±SE of at least three separate experiments. A total of 65 to 82 nuclei were counted for each observation. * denotes p<0.05 compared to controls while^ #^ denotes p<0.05, compared to the H_2_O_2_-treated group.

Finally, we performed TUNEL assay under the similar experimental conditions. Our data showed an increase of TUNEL positive nuclei in H_2_O_2_ treated cells as well as knockdown of catalase, Cu/Zn-SOD, and Bcl_2_. Representative of fluorescence microscopy images of TUNEL positive nuclei (FITC-positive) are shown in Figure 6 A and B. Transfection with scrambled si-RNAs followed by H_2_O_2_ treatment showed an increase of TUNEL positive nuclei from 3.7±0.3 to 18.2±0.4. Furthermore, knockdown of catalase, Cu/Zn-SOD and Bcl_2_ resulted in a significant increase in the TUNEL positive nuclei to 26.3±1.2% (p<0.05), 24.0±3.6% (p<0.05) and 30.7±0.9% (p<0.05), respectively (Figure 6 C), compared with the control cells. H_2_O_2_ treatment in the similar experimental set up (knocked down cells), resulted an additive effect and increased the TUNEL positive nuclei to 50.7±5.9% (p<0.05) for catalase, 46.3±6.6% (p<0.05) for Cu/Zn-SOD, and 58.7±10.4% (p<0.05) for Bcl_2_, respectively, compared to the scramble si-RNA (23.2±1.9%). Pretreatment with Tβ4 in the H_2_O_2_ treated group resulted in a significant reduction in the TUNEL-positive nuclei to 18.0±2.1% (p<0.05) in si-RNA-catalase, 18.7±2.0% (p<0.05) in si-RNA Cu/Zn-SOD, and 31.3±7.8% (p<0.05) in si-RNA Bcl_2_, respectively (Figure 6 C). These results indicate that Tβ4 selectively targets antioxidant and anti-apoptotic genes to provide protection under oxidative stress.

## Discussion

The current study was aimed to determine the target molecules modulated by Tβ4 that enables it to prevent oxidative stress in an *in vitro* system using cardiac fibroblasts. H_2_O_2_ induced a marked increase in intracellular ROS in the cardiac fibroblasts. This increase in the intracellular ROS led to altered expression of antioxidant enzymes like the SODs and catalase. Increased ROS also led to the loss of mitochondrial membrane potential and subsequently an increase in the Bax/Bcl_2_ ratio favoring apoptosis. This study shows for the first time that Tβ4 reduced the ROS accumulation by modulating the expression of antioxidant enzymes especially catalase and Cu/Zn-SOD and thereby preventing the loss of mitochondrial membrane potential. Additionally, Tβ4 upregulated the expression of anti-apoptotic protein Bcl_2_ and prevented apoptosis of cardiac fibroblasts in response to oxidative stress. Tβ4 failed to alleviate the oxidative stress and prevent cardiac apoptosis when these molecules were knocked down in the cells. Thus, Tβ4 possibly targets antioxidant and anti-apoptotic genes to provide protection under oxidative stress in cardiac fibroblasts.

The myocardium is composed of cardiomyocytes, fibroblasts, endothelial cells and other cell types along with surrounding extracellular matrix (ECM). Apart from the myocytes, cardiac fibroblasts make up the bulk of the myocardium and important cell type as they can play crucial role in cardiac protection by providing sustenance, ECM support and defense against various stress conditions. Therefore, in this study, we used cardiac fibroblasts, so as to study the effect of Tβ4 on this cell type and to study how oxidative stress affects them in the *in vitro* system.

In the cardiac cells, H_2_O_2_ is known to induce oxidative stress and apoptosis. This system has been used on various occasions as a model to study the regulation of stress signaling stimuli in cell death [37], [38]. Here, we used this model system to show the effects of Tβ4 on H_2_O_2_ induced fibroblast cell death. Our results indicate that 2 h pretreatment with Tβ4 is able to significantly decrease the apoptotic effects of H_2_O_2_ in cardiac fibroblasts and thus protecting from cell death. These results suggest that intracellular and biochemical effects stimulated by Tβ4 treatment play a crucial role in the cardio-protection under oxidative stress. H_2_O_2_ treatment on cardiac fibroblasts leads to increase ROS accumulation especially O_2_^.-^, H_2_O_2_ or OH^-^. Superoxide dismutase (SOD) converts O_2_^.-^ into H_2_O_2_ and the latter can generate hydroxyl (OH^-^) radicals in the presence of Fe^2+^ cations. ROS are able to oxidize biological macromolecules such as DNA, protein and lipids [39], [40], [41]. Also, NO can also be oxidized into reactive nitric oxide species, which may show behavior similar to that of ROS. In particular, the combination of NO and O_2_^.-^ can yield a strong biological oxidant, peroxynitrite that is more detrimental to the cells [42]. Oxidative stress causes altered expression of antioxidant enzymes SOD and catalase which prevents effective scavenging of the ROS formed as a result of H_2_O_2_ treatment in the fibroblasts. Our results show that Tβ4 restored the levels of Cu/Zn-SOD and catalase close to normal physiological level even under oxidative stress and thus scavenging the extra H_2_O_2_-induced ROS from the cellular system. One of the traditional hallmarks of ROS initiated cell death is mitochondrial dysfunction and energy depletion [43]. This is manifested by opening of the mitochondrial permeability transition pore (MPTP), the collapse of the mitochondrial membrane potential (Δψm) and a concomitant drop in ATP production [44]. These events lead to cascade of cell destruction and apoptosis. Increased production of ROS in the failing heart leads to mitochondrial permeability transition [45], which causes loss of mitochondrial membrane potential, swelling of mitochondrial matrix, release of apoptotic signaling molecules, such as cytochrome c, from the inter-membrane space, and irreversible injury to the mitochondria [46]. Increased ROS in our system led to a decrease in the Δψm as evident by the staining with MitoTracker Red. This loss of Δψm was prevented by pretreatment of Tβ4. At this point, it is beyond our scope to investigate how Tβ4 modulate mitochondrial membrane potential under oxidative stress and, therefore, warranted further investigation.

Tβ4 was extremely effective in reducing intracellular ROS in H_2_O_2_ treated cardiac fibroblasts (Figure 2 A-C). Tβ4 acts *via* upregulation of selected antioxidant genes like Cu/Zn-SOD and catalase. In fact, the intracellular ROS level was markedly reduced when the cells were pretreated with Tβ4 at least 2 h prior to H_2_O_2_ exposure, suggesting that it might activate the key molecules that play an important role in the enzymatic antioxidant defense system. Another particularly relevant protein that loses function upon oxidation is Mn-SOD; its loss of function would further compromise antioxidant capacity and lead to further oxidative stress [47]. Both Mn-SOD and Cu/Zn-SOD have been reported to play a crucial role in protecting the cardiac cells from oxidative damage by scavenging ROS [48]. In our experimental system, we found that Tβ4 upregulated the expression levels of Cu/Zn-SOD and not Mn-SOD in cardiac fibroblast thus affording cardiac protection which is in contrast to the previous report by Ho *et al* [23]. This could be probably due to the different cell type used in the study. Catalase, which was directly responsible for H_2_O_2_ clearance, was upregulated by Tβ4 both at protein and gene level in the presence of H_2_O_2,_ indicating that Tβ4 preferentially targets catalase which enables effecting scavenging of the H_2_O_2_ from the system. The mechanism of Cu/Zn-SOD and catalase upregulation by Tβ4 is currently unknown but, a transcription factor mediator activity has been postulated [49]. Tβ4 has been reported to translocate into the nucleus by an active transport mechanism or possibly through its cluster of positively charged amino acid residues (KSKLKK) but the exact function is still obscure. Alternatively, it might be the similar event like nuclear localization of actin where it is postulated that it might involve in chromatin remodeling [50], mRNA processing and transport [51].

Improved fibroblast survival during oxidative stress is important because increased fibroblast survival belittles the cardiac injury and prevents overall damage to the heart. Oxidative stress is known to trigger apoptotic cell death by up regulating the expression of pro-apoptotic protein like Bax which then dimerises and translocate to the outer mitochondrial membrane and aggregates to form pores that permit cytochrome *c* from mitochondria to cytosol [52]. Oxidative stress also leads to activation of caspase-3 cleavage and initiation of intrinsic apoptotic pathway [52]. Therefore, reduction in the production and accumulation of intracellular ROS is important to protect the cells from apoptosis triggered by the intrinsic pathway. Our data further showed that Tβ4 treatment inhibits excessive Bax expression and caspase-3 activation and enhance Bcl_2_ expression in fibroblast suggesting its protective effects under oxidative stress. As Tβ4 reduces the intracellular ROS levels, it would be reasonable to explain that Tβ4 probably promote the Bcl_2_/Bax ratio to protect the cells from apoptosis. To best of our knowledge, this is the first report that elucidates the beneficial effect of Tβ4 in cardiac fibroblast by up regulating the Bcl_2_/Bax ratio in favor of cell survival. This finding is in contrast to the previous report by Sosne *et al*, where they did not observe any change in the Bax/ Bcl_2_ expression [18]. This could be probably due to the different cell type used in the study. Although, there is no definitive mechanism by which Tβ4 exerts this anti-apoptotic effects, but, an internalization of Tβ4 has been proposed as one of the possible mechanism in human corneal epithelial cell under oxidative stress [19]. Alternatively, Tβ4 may use its methionine residues to react with intracellular oxygen at the post-translational level to generate sulfoxide, reducing the ROS level and eventually protects the cells from apoptosis [53].

Increased oxidative stress leads to pro-fibrotic changes in the fibroblasts [54], [55]. We observed that there was an increase in the expression of pro-fibrotic genes like CTGF, Collagen-I, Collgen-III and fibronectin in cardiac fibroblasts treated with H_2_O_2_. Decreased expression of fibrotic genes in the fibroblasts is beneficial because it prevents cardiac fibrosis and stiffening by reducing the ECM deposition. Our study showed that the increase in the pro-fibrotic gene expression was prevented by pretreatment of fibroblasts with Tβ4. These results corroborated with our previous observation in myocardial infarction (MI) model [22]. We showed that Tβ4 abrogated cardiac fibrosis in post-MI period by attenuating collagen type I and type III gene expression. This is the first report that elucidates the down regulation of pro-fibrotic gene expression by Tβ4 under oxidative stress.

Interestingly, we found that Tβ4 prevented apoptotic cell death by specifically targeting these antioxidant and anti-apoptotic molecules and when these antioxidant and apoptotic molecules were knocked down by si-RNA treatment; Tβ4 was not able to contribute its cardio-protective effects. These findings led us to suggest that Tβ4 provide cardiac protection by reducing the intracellular ROS levels and enhancing the expression of antioxidant enzymes (Cu/Zn-SOD and catalase) and anti-apoptotic protein (Bcl_2_) under oxidative stress.

In conclusion, we demonstrated that Tβ4 protects the cardiac fibroblasts against apoptosis by reducing intracellular oxidative stress through enhancing the expression of selected anti-oxidative enzymes and anti-apoptotic proteins. Given the preference of cardio-protective effects of Tβ4, it is still not clear how Tβ4 exerts its beneficial effects, is still under investigation. Although, Tβ4 is internalized by cells but the cell surface receptors are still not known. Furthermore, high concentration and ubiquitous presence of Tβ4 in the organs/tissues, it is reasonable to advocate that Tβ4 functions as an important intracellular mediator when either release from the cells or exogenously added, acts as a moonlighting peptide for repairing the damages tissues or cells [56], [57]. Our results not only offered more mechanistic explanation about the protective mechanism of Tβ4 but also supported the need to further investigate the use of this peptide in protecting the myocardium against oxidative damage in variety of disease condition where ROS has been implicated to play a damaging role. Also, further investigations are needed to explore the anti-fibrotic properties of Tβ4 which could be used to alleviate detrimental conditions like cardiac fibrosis, hypertrophy and heart failure.

### Clinical Implications

Many studies have shown that there is a depletion of anti-oxidant levels due to aging especially in the heart, which makes it more vulnerable to damage especially under ischemia and under high pro-oxidant condition. We believe that the identified molecules (Cu/Zn-SOD, catalase and Bcl_2_) are important players under oxidative stress to mitigate the damage. Our findings indicated that the beneficial role of Tβ4 and its effects on upregulation of anti-oxidant genes under oxidative stress in the cardiac fibroblasts might be of clinical relevance. This makes Tβ4 a better therapeutic target and could have wider implications once translated from bench to bedside.

### Limitation of the Study

In this study, we validated the effect of Tβ4 by selectively knocking down the anti-oxidant and apoptotic genes targeted by Tβ4 in cardiac fibroblasts. However, the possibility of interaction between these genes and other molecular pathways cannot be ignored. Also other off target effects of our anti-oxidative strategies with Tβ4 in conditions like cardiac fibrosis, cardiomyopathies and heart failure needs to be further investigated.
