# Correction: Thymosin Beta 4 Prevents Oxidative Stress by Targeting Antioxidant and Anti-Apoptotic Genes in Cardiac Fibroblasts

**DOI:** 10.1371/annotation/af7b47d5-5246-4e90-9691-f5894e119c60

**Published:** 2011-11-08

**Authors:** Sandeep Kumar, Sudhiranjan Gupta

The authors with to add the following funding source to their financial disclosure information: The work was partly supported by Scott & White RGP Award (R8000 R3536) to SG.

